# Determination of abnormally expressed microRNAs in bone marrow smears from patients with follicular lymphomas

**DOI:** 10.1186/2193-1801-3-288

**Published:** 2014-06-07

**Authors:** Yoshifumi Takei, Naomi Ohnishi, Mayumi Kisaka, Keichiro Mihara

**Affiliations:** Division of Disease Models, Center for Neurological Diseases and Cancer, Nagoya University Graduate School of Medicine, 65 Tsurumai-cho, Showa-ku, Nagoya, 466-8550 Japan; Department of Biochemistry, Nagoya University Graduate School of Medicine, 65 Tsurumai-cho, Showa-ku, Nagoya, 466-8550 Japan; Life Technologies Japan, 4-5-4 Hatchobori, Chuo-ku Tokyo, 104-0032 Japan; Department of Hematology and Oncology, Research Institute for Radiation Biology and Medicine, Hiroshima University, 1-2-3 Kasumi, Minami-ku Hiroshima, 734-8551 Japan

**Keywords:** MicroRNA (miRNA), Smears, Bone marrow, Quantitative PCR, Follicular lymphoma

## Abstract

The abnormal expression of microRNAs (miRNAs) is implicated in various human diseases, including cancers. Accordingly, miRNA expressions have been examined in many cancer tissues and blood, but there have been few studies examining smear samples from bone marrow (BM) or peripheral blood. Here we successfully isolated small RNAs from BM smears using a mirVana miRNA Isolation Kit with our original modifications. The isolated small RNAs were then used to measure the levels of representative miRNAs such as miR-155, let-7a, and U6 via real-time PCR with a specific TaqMan probe, although peaks for the ribosomal RNAs (18S, and 28S) were not identified. The PCR curves of the miRNAs were indistinguishable from those from BM living cells from the same donor. Finally, our method for BM smears identified numerous abnormally altered miRNAs (significantly decreased, 39 miRNAs; significantly increased, 27 miRNAs) in follicular lymphomas (FL) compared with normal donors via TaqMan real-time PCR miRNA array. The array indicated that miR-451 showed the greatest decrease in FL (a 345-fold decrease), while miR-338-5p showed the greatest increase in FL (172-fold) relative to normal donors. The miRNAs identified by our study might serve as markers to predict the invasion of FL cells into BM without biopsy. Furthermore, our method will provide a new avenue for the analysis of miRNAs in BM smear samples from various hematologic diseases.

**Electronic supplementary material:**

The online version of this article (doi: 10.1186/2193-1801-3-288) contains supplementary material, which is available to authorized users.

## Introduction

MicroRNAs (miRNAs), which are short non-coding single-stranded RNAs of 18–24 mer in length, negatively regulate the target gene expression at the post-transcriptional stage (Bartel 2004; Ambros 2004). miRNAs act to inhibit protein translation or degrade transcripts of the target gene (Bartel 2009). Many studies have demonstrated that abnormal expression of miRNAs is implicated in a variety of human diseases, including cancers (Calin and Croce 2006; Takei et al. 2011). Accordingly, the levels of various miRNAs have been measured in body fluids such as blood (serum or plasma), urine, cerebrospinal fluid, and ascites of patients with cancer (Cortez et al. 2011; Etheridge et al. 2011). Based on these measurements, some of the circulating miRNAs have been regarded as beneficial biomarkers to efficiently diagnose cancers. Recent reports have indicated that circulating miRNAs are quite stable even in an extracellular ribonuclease(s)-ubiquitous environment, such as in blood or other body fluids (Grasedieck et al. 2012), since most of the miRNAs there are packaged into exosomes or microvesicles (Hunter et al. 2008; Skog et al. 2008), into lipoprotein particles with high density (Vickers et al. 2011), or into apoptotic bodies (Zernecke et al. 2009). These molecular packaging mechanisms protect the miRNAs in body fluids from degradation, and thus, particularly in hematological cancers, some blood-circulating miRNAs can become excellent biomarkers to directly mirror the origin of cancers (Grasedieck et al. 2013).

Microscopic bone marrow (BM) examination is performed to diagnose many blood diseases, including leukemia, lymphoma, and multiple myeloma (Ryan 2010). The BM generates the cells in blood, including red blood cells, white blood cells, and platelets. BM samples are ordinarily acquired by aspiration or trephine biopsy, and much information can be derived from the blood cells in BM. Like peripheral blood smears, the BM samples are often stored as smears at room temperature (Miura et al. 2011). These smears also can be transported anywhere while remaining at room temperature, and thus are convenient for use in diagnoses.

Here we successfully isolated the small RNA fractions from BM smears, and showed that these fractions could be used to quantitate miRNAs via a typical real-time PCR method. In addition, we applied the small RNA fractions to a TaqMan real-time PCR miRNA array, and thereby identified many miRNAs whose levels were significantly altered in follicular lymphoma (FL), compared with the BM smears from normal donors. To our knowledge, this is the first report in which disease-associated miRNAs were identified from BM smears.

## Materials and methods

### Patients

In the present study, we studied bone marrow (BM) samples from patients with follicular lymphoma (FL) of B cell-type who were diagnosed at Hiroshima University Hospital, and from normal donors used as a control. All patients provided informed consent according to the Declaration of Helsinki. The study protocol was approved by the Institutional Review Board of Hiroshima University Hospital. All of the FL patients studied in the article were classified on Stage IV, and in all cases, the infiltration of the malignant cells into BM was observed (Table 1). The extent of BM infiltration of the malignant cells via flow cytometric analysis (Mihara et al. 2010) was also given in Table 1. For normal donors, we used the BM specimens from slight pancytopenia patients. On admission, although they were suspected to be aplastic anemia, we found they showed pancytopenia by individual differences according to our clinical examinations. Their pancytopenia was not progressive. Their BM was qualitatively and quantitatively normal without filtration of any abnormal cells.

**Table 1:** **A list of normal donors and FL patients with (B cell type) in the study**

ID	Age (years)	Gender	Clinical stage	The extent of BM infiltration by lymphoma cells	Date*	Age of the BM smears from the preparation up to use (Stock time at room temperature)
Normal 1	22	Male	-	0%	Jul 9, 2008	2 years and 10 months
Normal 2	43	Male	-	0%	Jul 19, 2006	4 years and 10 months
Normal 3	29	Male	-	0%	Apr 16, 2008	3 years and 1 month
FL Patient 1	61	Male	IVA	46%	Aug 11, 2006	4 years and 9 months
FL Patient 2	66	Male	IVA	54%	Aug 29, 2008	2 years and 9 months
FL Patient 3	72	Male	IVA	49%	Apr 5, 2006	5 years and 1 month
FL Patient 4	68	Male	IVA	38%	Sep 5, 2008	2 years and 10 months
FL Patient 5	59	Male	IVA	42%	Sep 15, 2006	4 years and 10 months

*Date of BM sampling and preparation of BM smears.

### Preparation of BM smears

BM samples were obtained via an aspiration and biopsy. BM smears were made by placing a drop of BM on a glass slide by the previously described method (Ryan 2010). Table 1 provides a list of BM smears from patients with FL (B cell type) and normal donors, along with the date of sampling and preparation of smears. We also showed each age of the BM smears (a stock time from the preparation up to use). All of the BM smears without fixation were kept at room temperature until use.

### Isolation of small RNAs from BM smears

Isolation of small RNAs, including miRNAs from BM smears, was performed using a mirVana miRNA Isolation Kit, or an RNAqueous-Micro Kit (a protocol for laser-captured microdissection samples) (Life Technologies Japan, Tokyo, Japan). We modified the manufacturer’s protocol for adequate isolation from BM smears.

For the isolation using the mirVana miRNA Isolation Kit, one BM smear slide was placed on a clean 100 mm dish, and lysis/binding buffer (300 μl) from the kit was dropped on the slide. Using a clean cell scraper and a pipette with a 1-ml tip, the dried smear component on the slide could be resolved but with high viscosity. The lysate from two BM smear slides was collected into a 2-ml tube, and the liquid volume was measured. A one-tenth volume (miRNA Homogenate Additive) of the lysate was added, and then the solution was mixed and incubated on ice for 10 minutes. An equal volume (acid-phenol chloroform) of the lysate was added, and then the solution was mixed with a vortex for 5 minutes, and centrifuged (10,000 rpm for 5 minutes). This extraction step can be repeated two or three times; the repetition was particularly effective for the already-stained BM smears. The water-phase was collected, and its liquid volume measured. Ethanol (a 1.25-times volume of the water-phase) was added, and the solution was mixed. All of the mixture was applied onto a micro-filter cartridge and centrifuged (the flow-through liquid was discarded). After washing the filter cartridge with Wash Solution 1 and 2/3, 100 μl of an elution solution heated at 95°C was added to the column, and 5 minutes later the desired solution was recovered by centrifugation.

For the isolation using the RNAqueous-Micro Kit, Lysis Solution (300 μl) from the kit was added onto one BM smear slide, and scraped using a clean cell scraper. For another BM smear slide, we repeated the procedure. The lysate (total 600 μl from two BM smear slides) was incubated at 42°C for 30 minutes, and then the supernatant by centrifugation was collected. Fifteen μl of LCM Additive from the kit was added to the supernatant (500 μl) and the solution was vigorously mixed, and then ethanol (1.25-times volume of the supernatant) was added with mixing. All of the mixture was applied onto a micro-filter cartridge and centrifuged (the flow-through liquid was discarded). After washing the cartridge with Wash Solution 1 and 2/3, 10 μl of an elution solution heated at 95°C was added to the column, and 5 minutes later the desired solution was recovered. The elution was repeated using another 10 μl of elution solution. Finally, 20 μl of the eluted solution was obtained.

Our modified methods are just matched for two BM smear slides (e.g., each liquid volume and handling, and column and tube size). The isolated small RNAs from two BM slides are enough to carry out several examinations such as qPCR, and array analysis. The critical points for the effective isolation are on ‘acid-phenol chloroform step’ to prevent and regulate high viscosity, which is peculiar to BM smears. The repetition of the step reduced their viscosity.

The RNA concentration of the eluted solution was determined by a NanoDrop ND-1000 spectrophotometer (Invitrogen), and its quality was examined using an Agilent 2100 Bioanalyzer (Agilent Technologies).

### Quantitative reverse transcription-PCR (qRT-PCR) for miRNA

Small RNAs isolated from BM smears were subjected to qRT-PCR for miRNAs such as U6, miR-155, and let-7a. Each RNA sample (10 ng) was reverse-transcribed using a TaqMan MicroRNA RT Kit and TaqMan MicroRNA Assay (Applied Biosystems), and the obtained cDNA was analyzed using specific TaqMan probes (U6, Assay ID 001973; miR-155, Assay ID 000479; and let-7a Assay ID 000377) and TaqMan Universal PCR Master Mix as described previously (Takei et al. 2011). All of the reactions and analyses were performed using a StepOne Real-Time PCR System (Applied Biosystems).

### TaqMan real-time PCR miRNA array

The RNA samples shown in Table 2 (from five patients with FL and three normal donors) were subjected to a TaqMan Array MicroRNA Card (version 2.0 for humans; Applied Biosystems). This real-time PCR miRNA array allows the simultaneous quantitation of up to 667 human miRNAs in a sample. For the RT reaction, we used the Megaplex RT Primers Pool for humans (Applied Biosystems), which is suitable for comprehensive miRNA measurement. Each RNA sample (500 ng) was thus subjected to both Megaplex RT reactions and subsequent PCR reactions according to the manufacturer’s protocol. All of the reactions were run on the ABI PRISM 7900 System (Applied Biosystems). Raw data were analyzed by using DataAssist software (version 3.0; Applied Biosystems). For the undetectable results on PCR, we defined the C_T_ value as 40. The expressions of all the miRNAs were calculated relative to that of U6 small nuclear RNA (RNU6B) by the comparative ΔCt method (Livak and Schmittgen 2001).

**Table 2:** **Quality check of the isolated RNAs from normal donors and FL patients according to our proposed method**

ID	RNA concentration (ng/μl)	Total yield (μg)	A260/A280	RIN*
Normal 1	27.6	2.8	1.81	2.5
Normal 2	28.2	2.8	1.87	2.6
Normal 3	33.8	3.4	1.91	2.6
FL Patient 1	24.6	2.5	1.74	2.6
FL Patient 2	28.0	2.8	1.84	2.5
FL Patient 3	14.4	1.4	1.88	2.4
FL Patient 4	17.9	1.8	1.82	2.6
FL Patient 5	22.5	2.3	1.80	2.5

*RNA integrity number.

### Statistical analysis

Statistical analysis on TaqMan real-time PCR miRNA array was performed using the Mann-Whitney U test. Probability values were shown in each table and the values less than 0.01 (1 × 10^-2^) were considered to indicate significant differences.

## Results

### Successful isolation of small RNAs from BM smears

**Figure 1:**
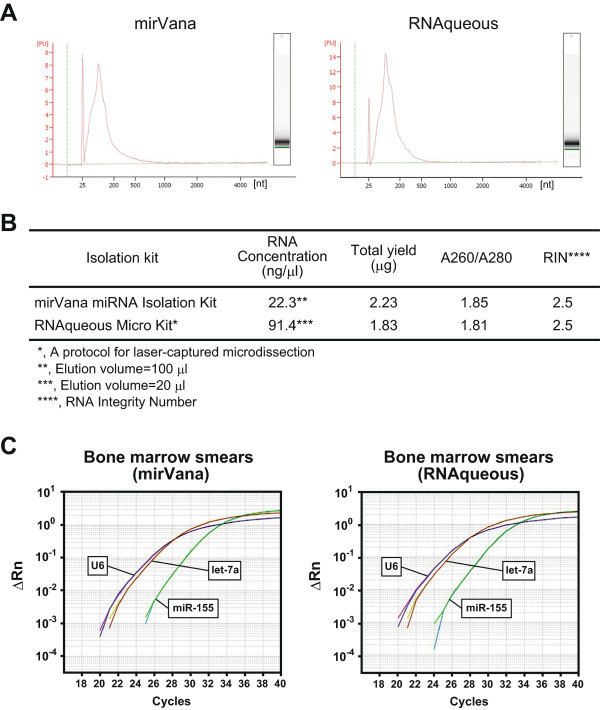
**Successful isolation of small RNAs from BM smears and functional quantitative PCR analysis for the miRNAs. A**, Ferrograms of the isolated RNA from BM smears. Left, mirVana miRNA Isolation Kit; and right, RNAqueous-Micro Kit. nt, nucleotide. **B**, Quality of the isolated small RNAs. RIN, RNA integrity number. **C**, Functional qRT-PCR for miRNAs (U6, let-7a, and miR-155).

We used two isolation kits with modifications to obtain small RNAs from BM smears: a mirVana miRNA Isolation Kit and an RNAqueous Kit. Both kits revealed very similar ferrograms (Agilent 2100 Bioanalyzer) of isolated RNAs: only one peak of small RNAs (under 500 nt) was observed, but no peaks of ribosomal RNA (18S and 28S) were seen (Figure 1A). We determined the RNA concentration with a NanoDrop ND-1000 spectrophotometer, and the ratio of A260/A280 showed a satisfactory value (Figure 1B). The RNA integrity number (RIN) was 2.5. There were no differences in results between the two kits. These findings suggested that, in the BM smears, the RNAs of larger size (over 500 nt) were completely degraded, whereas the smaller RNAs remained potentially reactive.

### Successful quantitative PCR analysis for miRNAs in small RNA fractions from BM smears

Using the isolated small RNA fractions, we performed quantitative PCR analysis for three representative miRNAs (U6, let-7a, and miR-155) with TaqMan probes. All three miRNAs were successfully amplified (Figure 1C), demonstrating that the miRNAs from BM smears can be quantified by PCR. The PCR curves were not notably different between the two kits, suggesting both small RNAs were equally functional. We decided to use the mirVana miRNA Isolation Kit for further study due to its simplicity.

### Similar results were obtained in living cells of BM

**Figure 2:**
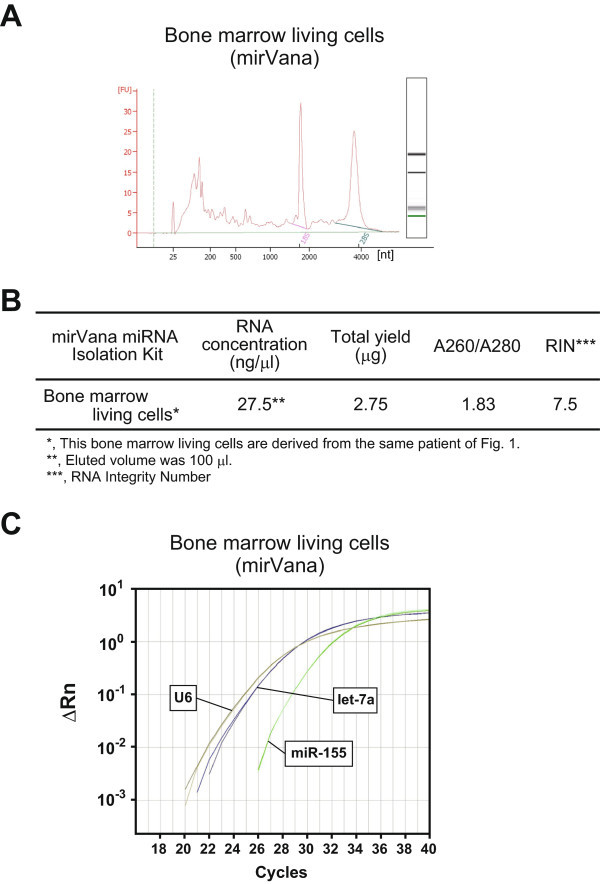
**Small RNAs from BM living cells gave almost identical results. A**, A ferrogram of the isolated RNA from BM living cells. nt, nucleotide. **B**, Quality of the isolated small RNAs. RIN, RNA integrity number. **C**, Functional qRT-PCR for miRNAs (U6, let-7a, and miR-155).

We obtained living cells of BM from the same donor who provided the BM smears. Figure 2A shows a ferrogram of BM living cells isolated by the mirVana miRNA Isolation Kit. Peaks of 18S and 28S were observed, and the RNA quality (A260/A280) was also satisfactory. The RIN was 7.5 (Figure 2B). Of course, the three miRNAs described above could be detected and quantified via the PCR method (Figure 2C). In view of the quantitative PCR analysis for the three miRNAs, the small RNA fraction from the BM smears was equivalent to that of the corresponding living cells (Figure 2C).

### The already-stained BM smears could also be used for quantitative PCR of the miRNAs

**Figure 3:**
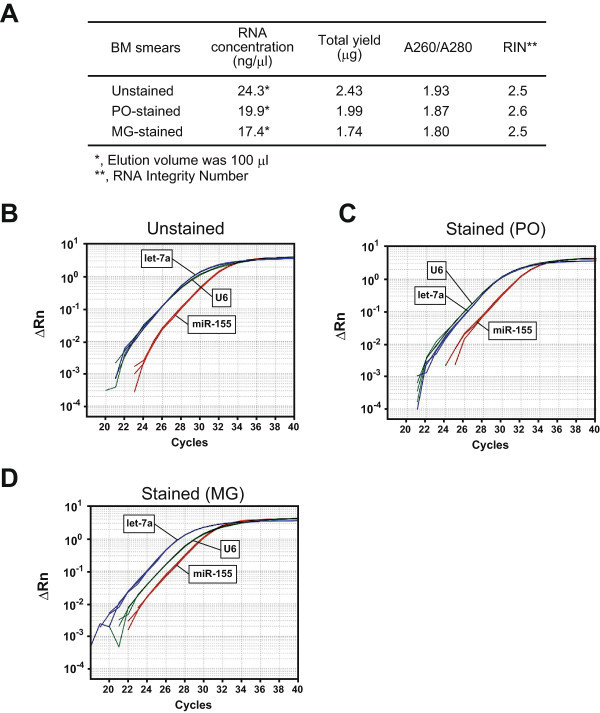
**Small RNAs from the already-stained BM smears could still be successfully used in qRT-PCR for miRNAs (U6, let-7a, and miR-155). A**, RNA quality, **B**, unstained; **C**, stained (PO); and **D**, stained (MG). PO, peroxidase staining; and MG, May-Giemsa staining.

We considered that our procedure would be more convenient if it could be applied to the already-stained smears, since the BM smears are often stained with peroxidase or May-Giemsa stain. Therefore, we isolated the small RNAs in the following three BM smears (unstained, PO-stained, and MG-stained) from the same donor. As shown in Figure 3A, the RNA quality (A260/A280) was not affected by the staining. The RIN was around 2.5 (Figure 3A). The quantitative PCR analysis for the three miRNAs (U6, let-7a, and miR-155) revealed that all three of the isolated small RNAs could be successfully used (Figure 3B-D). These results showed that small RNAs from BM smears, whether unstained or already-stained, can be used to quantitate miRNAs.

### miRNAs with significant abnormal alterations were found in the smears from the patients with FL compared with normal donors

Using our established isolation procedure for small RNAs, we quantitated the miRNA levels in BM smears from five FL patients and three normal donors. Tables 1 and 2 summarize the characteristics for each subject. The isolated small RNAs were subjected to the TaqMan real-time PCR miRNA array, and the results showed that the levels of many miRNAs were significantly different between the patients and the normal donors. The miRNAs whose levels were altered are shown in Table 3. The 20 miRNAs with the greatest increases and decreases are shown; the other significantly altered miRNAs are shown in Additional file 1. As shown in Table 3, the results of our analysis of abnormally expressed miRNAs in FL patients were surprising, since the differences between the abnormal miRNA expression levels and the expressions in normal donors were much greater than expected. For example, miR-451, the miRNA exhibiting the greatest decrease, was reduced by 345-fold in FL patients relative to normal donors; while miR-338-5p, the most-increased miRNA, showed a 172-fold increase. Among the decreased miRNAs (Table 3), miR-451 (Rank 1), miR-144 (Rank 2), and miR-144* (Rank 15) were clustered; and miR-452 (Rank 3) and miR-224 (Rank 5) were also clustered. With respect to the increased miRNAs, the three miRNAs (miR-24-2*, miR-23a, and miR-27a*) were registered in miRBase (Release 20, June 2013) as clustered miRNAs (Table 3). These results showed that abnormally expressed miRNAs often showed a tendency to gather and move together in groups (clusters), suggesting that there are some weak chromosomal region(s) in FL patients, such as chromosomal locations 17q11.2, Xq28, and 19p13.13 (Table 3).

**Table 3:** **Significantly altered miRNAs (Top 20) in the patients with FL, compared with normal donors**

Rank	miRNA	Clustered miRNAs	Fold change	Probability values*	Chromosomal location
Decreased					
1	miR-451	a	-345	2.6 × 10^-6^	17q11.2
2	miR-144	a	-179	1.6 × 10^-5^	17q11.2
3	miR-452	b	-140	1.7 × 10^-5^	Xq28
4	miR-494		-107	5.1 × 10^-5^	14q32.31
5	miR-224	b	-89.5	6.7 × 10^-5^	Xq28
6	miR-486-3p		-86.0	5.2 × 10^-5^	8p11.21
7	miR-483-3p		-75.1	4.5 × 10^-5^	11p15.5
8	miR-190a		-67.4	1.8 × 10^-4^	15q22.2
9	miR-10b*		-62.6	2.3 × 10^-4^	2q31.1
10	miR-939		-60.3	7.1 × 10^-5^	8q24.3
11	miR-1248		-55.8	1.1 × 10^-4^	3q27.3
12	miR-302b		-52.7	2.4 × 10^-4^	4q25
13	miR-1303		-50.9	5.1 × 10^-5^	5q33.2
14	miR-486-5p		-48.6	3.3 × 10^-4^	8p11.21
15	miR-144*	a	-45.5	2.2 × 10^-5^	17q11.2
16	miR-511		-41.2	3.4 × 10^-5^	10p12.33
17	miR-203a		-40.4	5.4 × 10^-5^	14q32.33
18	miR-202		-38.0	6.2 × 10^-4^	10q26.3
19	miR-204		-35.2	5.0 × 10^-5^	9q21.12
20	miR-182		-32.3	7.4 × 10^-4^	7q32.2
Increased					
1	miR-338-5p		172	3.2 × 10^-5^	17q25.3
2	miR-200a		75.9	6.4 × 10^-5^	1p36.33
3	miR-24-2*	c	45.4	6.0 × 10^-5^	19p13.13
4	miR-23a	c	41.2	4.9 × 10^-5^	19p13.13
5	miR-31		37.0	3.5 × 10^-4^	9p21.3
6	miR-639		35.9	5.9 × 10^-5^	19p13.12
7	miR-27a*	c	29.3	6.2 × 10^-4^	19p13.13
8	miR-29a*		26.9	5.8 × 10^-4^	7q32.3
9	miR-146b-3p		25.5	3.9 × 10^-5^	10q24.32
10	miR-374a*		23.0	3.2 × 10^-4^	Xq13.2
11	miR-766		21.1	5.3 × 10^-4^	Xq24
12	miR-1271		20.2	6.0 × 10^-5^	5q35.2
13	miR-181a-2*		19.3	5.4 × 10^-4^	9q33.3
14	miR-616		15.6	7.5 × 10^-5^	12q13.3
15	miR-7*		12.0	5.1 × 10^-5^	9q21.32
16	miR-30d*		10.3	8.8 × 10^-4^	8q24.22
17	miR-885-5p		10.2	2.9 × 10^-4^	3p25.3
18	miR-941		8.35	5.2 × 10^-4^	20q13.33
19	miR-1208		6.69	2.3 × 10^-4^	8q24.21
20	miR-26b*		6.57	2.0 × 10^-4^	2q35

a, b, and c: miRNAs clustered together in groups.

The same letter (a, b, or c) represents the miRNAs clustered in the same group.

*,Probability values by Mann-Whitney U test (VS normal donors) were shown.

Probability values less than 0.01 (1 × 10^-2^) were considered to indicate significant differences.

## Discussion

Smear samples are easy to store and to transport at room temperature. Thus, they are handy to use. They provide much information for the diagnosis of many diseases, including hematologic diseases, and particularly hematological malignancies. In the present study, we successfully isolated small RNAs from BM smears of FL patients, and according to TaqMan real-time PCR miRNA array, we also successfully determined many miRNAs that were significantly altered in the patients compared with normal donors (Table 3 and Additional file 1). To our knowledge, this is the first report in which disease-associated miRNAs were identified from BM smears.

The abnormal expression of miRNAs has been implicated in a variety of human diseases, including cancers. Circulating miRNAs have thus received much attention for their potential as stable blood-based biomarkers for detecting cancers Grasedieck et al. 2013; Mitchell et al. 2008; Hessvik et al. 2013). Circulating miRNAs in body fluids are much stable due to their packaging into exosomes, microvesicles, or lipoproteins (Hunter et al. 2008; Skog et al. 2008; Vickers et al. 2011). Regarding hematologic malignancies, the use of miRNA profiling for cancer biopsy samples from patients with FL has been reported (Roehle et al. 2008; Lawrie et al. 2009; Wang et al. 2012; Leich et al. 2011). However, circulating miRNAs in the BM of FL patients, especially in their BM smears, have not yet been reported. Based on a comparison of the previous reports (FL tissues; refs. Roehle et al. 2008; Lawrie et al. 2009; Wang et al. 2012; Leich et al. 2011) and our present report (BM smears from FL), the miRNAs that showed significant alterations in both cancer tissues and BM smears from FL compared with normal donors were as follows: the significantly decreased common miRNAs were miR-202 (Rank 18), and miR-139-5p (Rank 37); and the significantly increased miRNAs were miR-338-5p (Rank 1), miR-9 (Rank 21), and miR-330-3p (Rank 24). All of the other altered miRNAs in BM smears from FL patients, as shown in Table 3 and Additional file 1, were newly identified in our study.

FL is regarded as one of the malignant lymphomas that actively invade into BM; however, we currently have no ideas for appropriate marker(s) to detect such invasion without biopsy. Further, the mechanism(s) by which FL cells so readily invade into BM remains poorly understood. The results of the present study suggest that the measurement of some miRNA expression level(s) in BM smears can be used to correctively predict and diagnose FL without lymph node biopsy, although much investigation remains to be performed prior to the actual application for simple diagnosis of FL.

The stability of miRNAs in formalin-fixed paraffin-embedded (FFPE) tissues has already been reported, and thus many studies have shown that archived FFPE tissue samples can be used for PCR-based miRNA analysis (Li et al. 2007; Xi et al. 2007; Doleshal et al. 2008; Kolbert et al. 2013), or next-generation sequencing (Kelly et al. 2013; Meng et al. 2013). Here, the RIN of RNA fractions isolated from FFPE tissues was generally low (approximately 2.4–2.6), indicating that the RNAs were heavily fragmented. This, in turn, means that the small RNAs isolated from BM smears and FFPE tissues are quite similar to each other, and they might be suitable for miRNA quantitation rather than gene expression analysis. Indeed, our BM smears are not suitable for determining mRNA expression levels via quantitative RT-PCR (data not shown).

## Conclusion

Our isolation method provides a new avenue for the analysis of miRNAs in BM smear samples from patients with various diseases, particularly blood malignancies. Using this method, numerous miRNAs with potential as diagnostic markers in BM may be identified in the near future.

## Electronic supplementary material

Additional file 1: **Significantly decreased and increased miRNAs (below Rank 21) in FL patients, compared with normal donors.** (XLSX 13 KB)
